# A Portable Impedance Immunosensing System for Rapid Detection of *Salmonella Typhimurium*

**DOI:** 10.3390/s17091973

**Published:** 2017-08-28

**Authors:** Tao Wen, Ronghui Wang, America Sotero, Yanbin Li

**Affiliations:** 1 School of Mechanical and Electrical Engineering, Central South University of Forestry and Technology, Changsha 410004, China; wt207@sina.com; 2 Department of Biological and Agricultural Engineering, University of Arkansas, Fayetteville, AR 72701, USA; rwang@uark.edu (R.W.); asotero@email.uark.edu (A.S.)

**Keywords:** immunosensor, portable, label-free, *Salmonella Typhimurium*, rapid detection

## Abstract

*Salmonella* *Typhimurium* is one of the most dangerous foodborne pathogens and poses a significant threat to human health. The objective of this study was to develop a portable impedance immunosensing system for rapid and sensitive detection of *S*. *Typhimurium* in poultry. The developed portable impedance immunosensing system consisted of a gold interdigitated array microelectrode (IDAM), a signal acquisitive interface and a laptop computer with LabVIEW software. The IDAM was first functionalized with 16-Mercaptohexadecanoic acid, and streptavidin was immobilized onto the electrode surface through covalent bonding. Then, biotin-labelled *S*. *Typhimurium*-antibody was immobilized onto the IDAM surface. Samples were dropped on the surface of the IDAM and the *S*. *Typhimurium* cells in the samples were captured by the antibody on the IDAM. This resulted in impedance changes that were measured and displayed with the LabVIEW software. An equivalent circuit of the immunosensor demonstrated that the largest change in impedance was due to the electron-transfer resistance. The equivalent circuit showed an increase of 35% for the electron-transfer resistance value compared to the negative control. The calibration result indicated that the portable impedance immunosensing system could be used to measure the standard impedance elements, and it had a maximum error of measurement of approximately 13%. For pure culture detection, the system had a linear relationship between the impedance change and the logarithmic value of *S*. *Typhimurium* cells ranging from 76 to 7.6 × 10^6^ CFU (colony-forming unit) (50 μL)^−1^. The immunosensor also had a correlation coefficient of 0.98, and a high specificity for detection of *S*. *Typhimurium* cells with a limit of detection (LOD) of 10^2^ CFU (50 μL)^−1^. The detection time from the moment a sample was introduced to the display of the results was 1 h. To conclude, the portable impedance immunosensing system for detection of *S*. *Typhimurium* achieved an LOD that is comparable with commercial electrochemical impedance instruments. The developed impedance immunosensor has advantages in portability, low cost, rapid detection and label-free features showing a great potential for in-field detection of foodborne pathogens.

## 1. Introduction

*Salmonella Typhimurium* is one of the most dangerous foodborne pathogens and poses a significant threat to human health. *Salmonella* is generally transmitted to humans through the consumption of animal-related agro-products such as poultry, meat, eggs and milk [1]. The US Centers for Disease Control and Prevention (CDC) reported that *Salmonella* is estimated to cause one million foodborne illnesses in the United States, with 19,000 hospitalizations and 380 deaths annually [2]; therefore, there is an urgent need for the development of a rapid and reliable device to detect the presence of *Salmonella* in agricultural and food products.

Traditional methods for detection of *Salmonella* are dependent on microbiological methods, which include pre-enrichment steps, cultivation of bacteria, and serological validation of suspicious colonies [3]. Although these approaches usually provide reliable results, they are time-consuming (18 h for *S. Typhimurium*) and labor-intensive because they require multiple steps for the enrichment and growth of the bacteria [4]. Due to these drawbacks, new rapid methods have been developed over the past several decades and have been gradually used for detection of *Salmonella* and other foodborne pathogens. Polymerase chain reaction (PCR)-based methods have been shown to significantly lower the limit of detection to 5 CFU (colony forming unit) mL^−1^, but these approaches still require long and often complicated preparation steps, such as cell analysis and DNA separation [5,6]. Immunological assays such as ELISA can detect *Salmonella* at levels of 10^4^–10^5^ cells mL^−1^; and lower detection levels can be achieved by coupling ELISA methods with an enrichment step that usually takes between 16 and 24 h [7,8]. Despite significant advances, current *Salmonella* detection methods are neither fast, reliable, nor sensitive enough, nor are they easy to perform [9].

In recent years, biosensors have played an important role in the detection of pathogens in foods. There have been numerous detection methods developed over the past several decades that can detect foodborne pathogens effectively such as the electrochemical [10,11,12], quartz crystal microbalance [13,14,15], and optical methods [16,17,18]. An impedimetric biosensing technique, an electrochemical method, offers several advantages such as good sensitivity, miniaturization potential, and mass production [19]. It has also been proved to be very promising for rapid detection of foodborne pathogens, especially for on-site detection.

A key component of the impedimetric biosensor is the biological recognition element used for detection of specific targets. The design of the biological recognition element generally focuses on either indirect or direct detection. Indirect detection uses a sandwich-like structure comprising of the target recognition element that binds to the surface of the target, usually a bacterial cell, and the biochemical label that triggers a reaction of the chemical in the media. Direct detection, on the other hand, works by binding the target bacterial cells onto the surface of the biosensor [20]. The indirect detection method also requires the binding of two ligands to the target bacteria. This requirement prolongs the detection time making this method unsuitable for rapid on-site detection. Xu et al. (2016) developed an immunosensor using magnetic beads-antibodies (MBs-Ab) conjugates labeling for detection of *E. coli* O157:H7 and *S*. *Typhimurium* in foods [21]. This sensor could detect both bacteria at concentrations of 10^3^ CFU mL^−1^ in 2 h without an enrichment process. In comparison, direct detection methods bypass the labeling procedure that is normally required by other electrochemical biosensors and greatly shortens the detection time. Wang et al. (2015) developed an impedance immunosensor using magnetic nanobeads and screen-printed interdigitated electrodes for the rapid detection of *E. coli* O157:H7 [22]. The developed immunosensor lowered the detection time to under an hour for concentrations of 10^4^ CFU mL^−1^. However, when using a direct method, the immunosensors have relatively higher limit of detection (LOD) due to lacking additional amplification. Moreover, the consistency of the fabricated biosensors is greatly affected by the surface condition of the electrodes and the unspecific absorption of compounds in non-target samples.

At present, numerous impedimetric biosensor applications for detection of foodborne pathogens are implemented by commercial electrochemical instruments. The instruments use full-bands spectroscopy to detect the impedance change of the biosensor caused by specific binding. However, the data that is collected with impedance spectroscopy cannot be directly used for detection analysis since it contains data redundancies that result in low efficiency. Commercial instruments are expensive due to lack of pathogen specificity. In addition, commercial electrodes are commonly used in the sensor system and there is no customized design and integration of the components into a miniaturized potable device. All of these drawbacks have limited the use of impedimetric biosensors for in-field detection of detection of foodborne pathogens.

In our previous study, research was conducted to develop a portable impedance biosensor for detection of avian influenza (AI) virus [23]. In this study, we explored a new application of the potable impedance biosensor for detection of *S*. *Typhimurium* bacteria. However, the biosensor had to be redesigned since there are many differences in biological components, physical structure, binding sites, etc. between AI virus and *Salmonella* cells. One difference is size. The size of an AI virus particle is about 80–120 nm in diameter, while a rod-shaped *S. Typhimurium* cell, is about 1 µm long and 0.5 µm wide. The redesigned portable impedance immunosensing system utilized interdigitated array microelectrodes (IDAM) and label-free recognition methods for detection of *S*. *Typhimurium*. The system was designed for on-site detection of *Salmonella* in foods, which consisted of the impedance acquisition circuit and LabVIEW virtual instrument (2012, National Instrument, Austin, TX, USA). The IDAM was first functionalized with 16-Mercaptohexadecanoic acid, and streptavidin was conjugated through covalent bonding. Afterwards, biotin-labelled *S. Typhimurium*-antibody was immobilized onto the IDAM surface using biotin-streptavidin chemistry. The electrical characteristics of the immobilized IDAM were studied in detail using electrochemical analysis. The developed immunosensing system was first calibrated using standard resistors and capacitors, and then it was applied to pure culture and poultry samples to evaluate its feasibility, specificity and sensitivity for detecting *S*. *Typhimurium*.

## 2. Materials and Methods

### 2.1. Biological and Chemical Materials

Stock phosphate buffered saline (PBS, 0.1 mol L^−1^, pH 7.4) and bovine serum album (BSA) were purchased from Sigma-Aldrich (St. Louis, MI, USA). Stock PBS solution was diluted at a ratio of 1:10 to prepare 1 × PBS (10 mmol L^−1^, pH 7.4), and used for all the tests. In addition, 1% BSA solution (wt/vol) prepared in PBS was used as a blocking buffer. Ultrapure deionized water (18.2 MΩ cm) was obtained from Millipore (Milli-Q, Bedford, MA, USA). Monoclonal anti-*S. Typhimurium* antibodies (4.0–5.0 mg mL^−1^) were purchased from Meridian Life Science Inc. (Memphis, TN, USA). Furthermore, a 1:5 dilution of antibody (0.8–1.0 mg mL^−1^) was prepared with PBS and stored at 4 °C for further use.

### 2.2. Bacteria Culture and Surface Plating Method

*Salmonella Typhimurium* (Loeffler) Castellani and Chalmers (American Type Culture Collection-ATCC 14028) were obtained from the American Type Culture Collection (Rockville, MD, USA). The pure culture of *S*. *Typhimurium* was inoculated in brain heart infusion broth (Remel, Lenexa, KS, USA) and incubated at 37 °C for approximately 18 h [24]. The culture was serially diluted to 10^−7^ using a PBS buffer solution. Surface plate counting of the *S*. *Typhimurium* cells was performed by plating 0.1 mL of decimal dilutions on MacConkey sorbitol agar (Remel) plates. After incubation at 37 °C for 24 h, *S*. *Typhimurium* colonies on the plates were counted to determine the number of cells in CFU mL^−1^. For safety reasons, the culture was heat-killed in a boiling water bath for 30 min before being used in tests. Other non-target bacteria were also obtained from the American Type Culture Collection (Rockville, MD, USA) with detailed information provided in the Supplementary Material (Table S1).

### 2.3. Preparation of Poultry Samples

The detection of *S*. *Typhimurium* in chicken carcass rinse water was examined. The chicken carcass used to prepare the rinse water was a whole bird purchased from a supermarket. The rinse water used for detection was prepared using a suggested method from the U.S. Department of Agriculture Food Safety and Inspection Service [25]. The bird, weighing approximately 4.5 kg, was aseptically transferred into a large stomacher bag. In addition, 400 mL of 0.1% buffered peptone water (BPW) was poured into the stomacher bag. The stomacher bag was then shaken using rocking motions for about a minute. After shaking, the rinse water solution was collected and then it was filtered (pore size of 5 µm) using a syringe filtration.

One milliliter of killed *S*. *Typhimurium* cells at a concentration of 10^9^ CFU mL^−1^ was added to 9 mL of chicken rinse water to obtain the poultry samples contaminated with *S*. *Typhimurium*. The contaminated rinse water solution was further diluted to a concentration of 10^5^ CFU mL^-1^. All solution samples were used immediately in tests after collection.

### 2.4. Interdigitated Array Microelectrodes and Surface Modification

In order to improve the sensitivity of detection, the fabrication dimensions of the IDAM (Institute of Semiconductor of Chinese Academy of Science, Beijing, China) contained 25 pairs of digit microelectrodes with 15 µm digit width, 15 µm inter-digit space and 3 mm digit length. Prior to antibody immobilization, the IDAM was pretreated with 1 M NaOH for 30 min and 1 M HCl for 5 min in sequence to remove surface oxide. The IDAM was then rinsed with deionized water, dried under a stream of nitrogen, and then functionalized with 20 mM 16-mercaptohexadecanoic acid (MHDA) ethanol solution for 24–48 h at room temperature in the dark to form self-assembled monolayers (SAMs) on the surface of gold electrode. After the 24–48 h period, the IDAM was rinsed by spraying ethanol and deionized water successively and dried in a stream of nitrogen. Then, it was immersed in EDC (*N*-(3-dimethylaminopropyl)-*N*’-ethylcarbodiimide hydrochloride)/NHS (N-hydroxysuccinimide) (75 mM/30 mM, *v*/*v*, 1:1) solution at room temperature for 10 min to active its surface. Following surface activation, the IDAM was rinsed with deionized water, and then a 50 µL of streptavidin (1 mg/mL) was dropped onto the electrode surface and incubated for 40 min at room temperature. Following the incubation, the IDAM was rinsed with deionized water and dried in a stream of nitrogen. Then, a 50 µL of biotinlated antibody (0.8–1 mg mL^−1^) was dropped onto the electrode surface and incubated for 40 min at room temperature. After rinsing with deionized water and dried with nitrogen, a 50 µL of BSA (1% in PBS) was dropped onto the electrode surface and incubated for 30 min at room temperature. BSA is a commonly used blocking agent, and it was applied here to block uncoated surface sites and reduce non-specific adsorptions. Finally, the IDAM was rinsed with deionized water and dried under a stream of nitrogen, and it was ready for bacteria detection. For all the rinsing steps, a rinsing bottle was applied to spray deionized water for 20–30 s.

### 2.5. SEM Images

Scanning electron microscopy (SEM) with a high-resolution scanning electron microscope FEI Nova NanoLab 200 (FEI Company, Hillsboro, OR, USA) in field immersion mode at 15 kV accelerating voltage was used to observe the binding of antibody-bacteria complexes on the IDAM surface.

### 2.6. Principle of the Immunosensor

**Figure 1:**
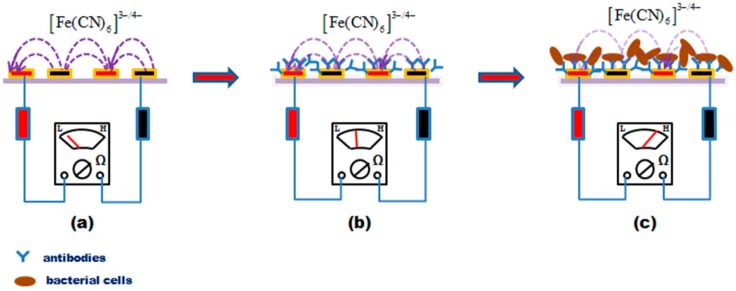
Principle of the immunosensor: (**a**) bare IDAM; (**b**) IDAM with antibody immobilization; and (**c**) IDAM with bind bacterial cells.

The principle of the immunosensor is shown in Figure 1. It is based on measurements of electrochemical Faradaic impedance in the presence of [Fe(CN)_6_]^3−/4−^ as a redox probe. As shown in Figure 1a, when a bare gold IDAM is immersed into an electrolyte solution containing the redox couple and a small-amplitude ac potential (5 mV) is applied to the electrode, the Faradaic process of oxidation and reduction of the redox couple occur. Electrons are then transferred between the two sets of array electrodes through the redox couple [26]. When antibodies are immobilized onto the electrode surface (Figure 1b), they form a layer that inhibits the electron transfer between the electrode and an increase in the electron transfer resistance should be expected. It is reported that the membranes of natural biological cells (thickness 5–10 nm) have a capacitance between 0.5–1.3 µF cm^−2^ and a resistance of 10^2^ to 10^5^ Ω cm^2^ [27]. If bacterial cells attach to the electrode surface (Figure 1c), the formation of antibody–antigen complexes caused by the immuno-reaction create a barrier that prevents the redox probe from making contact with the electrode surface, resulting in an increase in the electron-transfer resistance. The magnitude of the increase in electron-transfer resistance is related to the number of bacterial cells captured by the immobilized antibodies.

### 2.7. Design of Impedimetric Measurement Circuits

Theoretically, a label-free immunosensor can be treated as an unknown impedance component consisting of the electrolyte solution resistance (*R_s_*), Faradaic electron-transfer resistance (*R_et_*), dielectric capacitance of solution (*C_di_*) and double layer capacitance (*C_dl_*). Impedance is an expanded concept of resistance. It can be expressed as a complex number with a frequency independent part called real part *R* and a frequency dependent part called imaginary part *X*. A Bode plot showing the log of frequencies (*x*-axis) versus the log of impedance magnitudes and phase angle shift (*y*-axis) is used for impedance data presentation. The following formulas show the basic calculation in impedimetric measurement. The first is the calculation for the immunosensor impedance magnitude using Equation (1):

(1) $$\left|Z\right|=\sqrt{\left[R^{2}+{\left(X_{L}-X_{C}\right)}^{2}\right]}$$

where *R* is the resistance, *X_L_* is the impedance due to inductance, which is negligible in electrochemical measurement, and *X_C_* is the impedance due to capacitance. *X_C_* can be calculated using Equation (2):

(2) $$X_{c}=\frac{1}{2\pi fc}$$

where *f* is the frequency in Hz and *c* is the value of capacitor in F. The phase angle is calculated with Equation (3):

(3) $$\varphi =\tan^{-1}\left(\frac{X_{L}-X_{C}}{R}\right)$$

**Figure 2:**
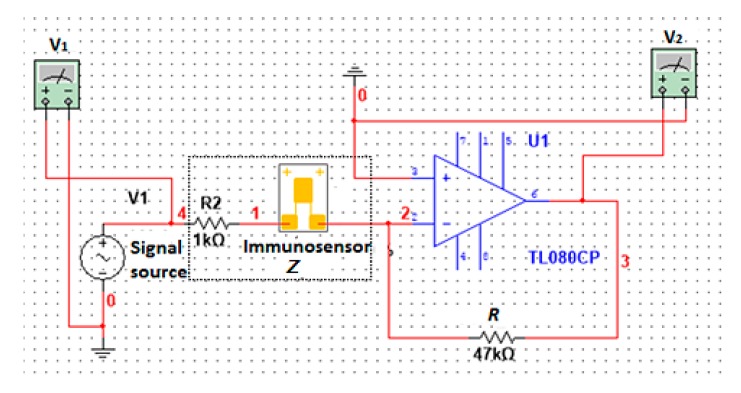
The circuit of impedance measurement for the immunosensor.

The impedance measurement uses a sinusoidal voltage within a certain frequency range as exciting signals [28]. According to the principles above, the impedance measurements taken using an immunosensor can be illustrated with the circuit in Figure 2.

The signal source power that was added to the series circuit consisted of a constant resistance *R* and an unknown component *Z*. When values of the voltage *V*_1_ and *V*_2_ were measured, the impedance *Z* of the unknown component was calculated using Equation (4). The relevant circuit of the impedance measurement is shown in Figure 2:

(4) $$Z=\frac{V_{1}}{V_{2}}R$$

### 2.8. Setup of the Portable Impedance Immunosensing System

**Figure 3:**
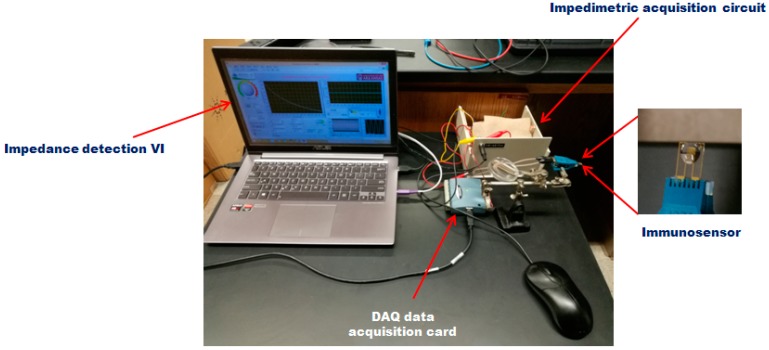
Setup of the portable impedance immunosensing system.

Figure 3 shows the setup of the portable impedance immunosensing system. The immunosensor was fabricated by immobilizing biotin labeled anti-Salmonella biomaterials with streptavidin-biotin method on the IDAM. The IDAM was mounted face-up on electrode holder. The data acquisition card (DAQ) data acquisition card (USB-1208 plus, Measurement Computing Corp., Norton, MA, USA) was served as a bridge for communication between the laptop and the impedimetric acquisition circuit. Measured data were collected by the impedimetric acquisition circuit and sent through the DAQ to the laptop. Scheme S1 (provided in Supplemental Materials) shows the scheme of the portable impedance immunosensing system. The sinusoidal voltage within a certain frequency range as an exciting signal source was generated by the LabVIEW program through the sound card of the laptop. When the exciting signal source was added to the impedimetric acquisition circuit, the impedance amplitude of the IDAM was calculated by measuring values of the voltage *V*_1_ and *V*_2_ through the DAQ card. The process of impedance measurement was controlled by the LabVIEW program.

### 2.9. Immunosensing Detection

The detection of *S. Typhimurium* bacteria was measured using a laptop with LabVIEW software. The structure and function of the software was improved using modular programming based on a separate virtual instrument (VI) system developed in our previous study [29], which consisted of audio signal generator, sine signal output adjustment, DAQ data acquisition and waveform display. The typical procedure of immunosensing detection for *S. Typhimurium* included: (1) dropping 50 µL of redox probe solution at the surface of the interdigitated array microelectrode (IDAM), then waiting for 1 min to obtain a stable baseline; (2) washing the redox solution off the surface of the IDAM using PBS buffer solutions; (3) dropping a 50 µL sample solution containing *S. Typhimurium* at the surface of the IDME, then inoculating for 40 min to allow the binding reaction between the antibodies immobilized on the IDAM and the target bacteria in sample solution; (4) repeating step 2 to wash off the sample solutions; and (5) repeating step 1 to measure the impedance of the IDAM in the presence of the redox probe. The change of impedance was correlated to the concentration of *S. Typhimurium* in sample solution.

### 2.10. Statistical Analysis

Since the baseline drifted each time, it was measured due to the fabrication qualify of the IDAM, the relative impedance change in percentage was calculated using Equation (5) to minimize the variation:

(5) $$Z_{R}=\frac{Z_{S}-Z_{B}}{Z_{B}}\times 100\%$$

where *Z_R_* is relative impedance change in percentage, *Z_s_* is impedance value caused by target samples, and *Z_B_* is impedance value after BSA blocking. The means ± standard deviations of quadruplicates were calculated using Excel 2013 software (Microsoft, Redmond, WA, USA). The statistical differences were determined with paired samples *t*-testing using SPSS 18.0 software (SPSS Inc., Chicago, IL, USA). The significance level was set at a *p*-value of <0.05.

## 3. Results and Discussion

### 3.1. Equivalent Circuit Simulation of the Immunosensor

**Figure 4:**
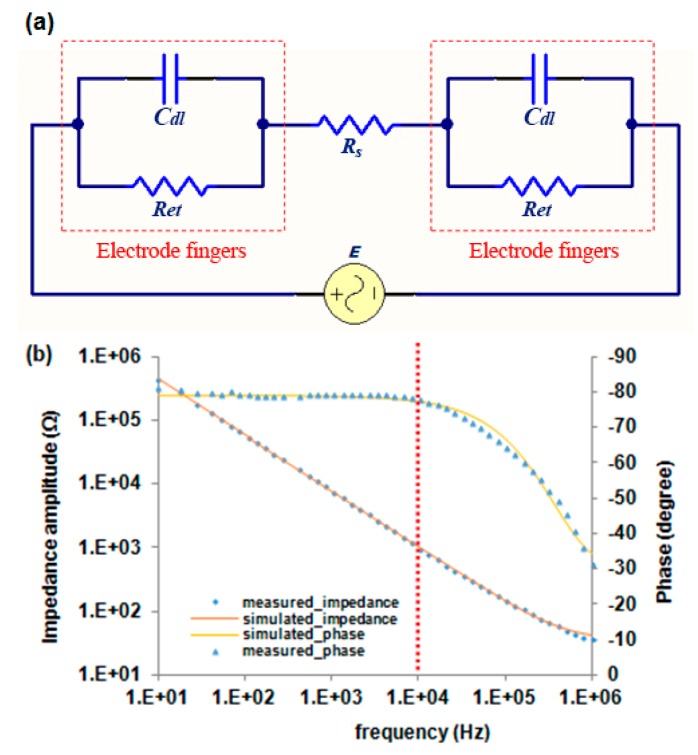
(**a**) The equivalent circuit of the immunosensor for detection of *S*. *Typhimurium*, and (**b**) Bode diagrams of the experimental impedance spectra and fitted curve data within a frequency range of 10 Hz to 1 MHz. The spectrum was recorded in the presence of PBS buffer containing 10 mM [Fe(CN)_6_]^3−/4−^ (1:1). The dashed line indicates the experimental data and the solid line indicates the fitted curve.

The performance of the immunosensor with two electrodes can be represented with an equivalent circuit that mimics the behavior of the cell under the same excitation conditions. Data from the electrochemical impedance spectra can be simulated using the equivalent circuit. The equivalent circuit was generated and validated using an IM-6 impedance analyzer with the IM-6/Thales 2.49 software (BAS, West Lafayette, IN, USA). The equivalent circuit of the electrochemical cells consists of two branch circuits (positive electrode and negative electrode) and an electrolyte solution that acts as a resistor (*R_s_*) between the two branch circuits. Each branch circuit includes a double layer capacitor (*C_dl_*), and electron-transfer resistance (*R_et_*). *C_dl_* represents the double layer capacitance of an electrode, accounting for the effect of ionic species on the capacitance near the surface of an electrode. *R_et_* represents the hindering access of the redox probe to the electrode surface in the Faradaic process and *R_s_* accounts for the change in conductivity of the bulk medium and charge transport across the bulk solution. The equivalent circuit is illustrated in Figure 4a.

The electrolyte solution (*R_s_*) and the two branch systems are connected in series since the current must pass through both of them. In each branch circuit, the electron-transfer resistance (*R_et_*) is connected in parallel with the double layer capacitance (*C_dl_*) because the total current through the electrode surface is the sum of Faradaic current (*i_e_*) and double layer current (*i_c_*). Figure 4b (dash line) shows the Bode diagram impedance spectrum of the antibody immobilized IDAM sensor after BSA blocking. The impedance was measured in a PBS buffer containing 10 mM [Fe(CN)_6_]^3−/4−^ (1:1). Fifty points of the measured data on the impedance spectrum were used as input to the equivalent circuit, generating a fitting impedance spectrum (Figure 4b solid line). Using this simulation, the values of *C*_dl_, *R_et_*, and *R_s_* were 1.87 nF, 15.08 kΩ and 782 Ω, respectively. The mean modulus impedance error and the phase angle error were 0.8% and 0.3, and the maximum error of the modulus impedance and the phase angle were 11.1% and 3.7, respectively. The agreement between the measured data and the fitted spectra indicted that this equivalent circuit was an accurate representation of the immunosensor. The impedance spectrum can be divided into two distinct regions the phase angle spectrum (Figure 4b). An electrical current passing through a capacitor is phase shifted by −90° with respect to the applied voltage and the phase angle in the capacitance dominated region is therefore close to −90°. The current passing through a resistor is always in phase with the applied voltage and the phase angle in a resistance-dominated region is close to 0°. As shown in Figure 4b, the phase angle is close to −80° in the low frequency range and decreases to 0° in the high frequency range. It can be concluded that the double layer capacitance and the electron-transfer resistance around each electrode are resistive at frequencies of 10 Hz to approximately 10 kHz, making the impedance value very high. The medium resistance could be ignored in this case. When the frequency is greater than 10 kHz (in the high frequency range), the phase angle decreases from −80° to 0° indicates that the medium resistance that contributes to the total impedance is amplified significantly.

### 3.2. Impedance Analysis of S. Typhimurium Captured on the Surface of Electrodes

**Figure 5:**
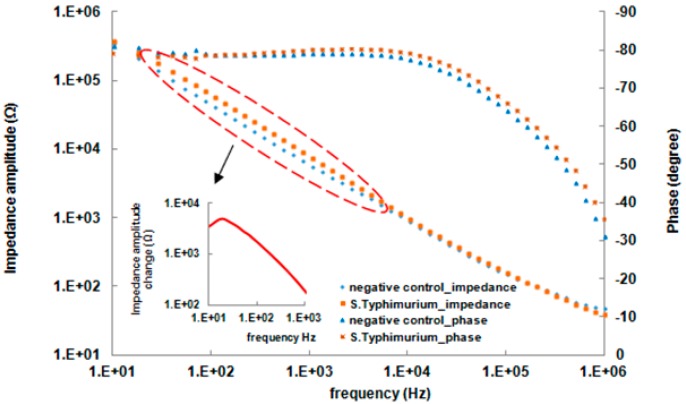
A typical Bode plot of measured impedance data of the negative control and *S*. *Typhimurium* sample at a concentration of 2.5 × 10^3^ CFU (50 µL)^−1^.

Impedance magnitudes and phase angles for detection of a sample containing 10^3^ CFU (50 µL)^−1^ *S. Typhimurium* and a negative control sample are shown in Figure 5. The presence of bacteria resulted in an increase in the impedance magnitude at a scanning frequency from 1 Hz to 1 MHz compared to negative control sample. The significant difference in impedance between the bacterial and control samples was observed below 1 kHz but was negligible at frequencies higher than 1 kHz. The phase angle describes the contribution of the resistance and capacitance elements to the impedance value. As shown in Figure 5, the capacitance and the resistance element dominate at different frequencies. From 10 Hz to 10 kHz the impedance value decreases, while the phase angle stays constant at −80°. From 10 kHz to 1 MHz the impedance value continues decreasing, whereas the phase angle decreases from −80° to 0°. This indicates that impedance measurement for the detection of bacteria can only be carried out by observing the behavior of the electrode surface at low frequencies or the medium at high frequencies. In order to better understand the effect that captured *S. Typhimurium* has on the impedance change of the electrode surface, the measured data from S. *Typhimurium* and the negative control were simulated using the equivalent circuit in Figure 4a. The values obtained from each element of the equivalent circuit are shown in Table S2 (provided in Supplemental Materials).

As shown in Table S2, the value of electron-transfer resistance increased from 15.08 to 20.45 kΩ due to the presence of *S*. *Typhimurium* cells, showing an increase of 35.6% over the electron-transfer resistance value for the negative control sample. The results demonstrated that the binding of *S. Typhimurium* cells to the antibody-immobilized electrode surface created a barrier for the electrochemical process, and the access of the redox probe was hindered to some degree, resulting in an increase in the *R_et_*. The value of the double layer capacitance due to the presence of *S. Typhimurium* cells increased from 1.87 to 2.11 nF, causing an increase of 12.9%. Theoretically, the value of the double layer capacitance mainly depends on the dielectric permittivity of the double layer, electrode layer covering area, and the thickness of the layer [30]. When *S. Typhimurium* cells attach to the electrode surface, the dielectric permittivity is enhanced and the double layer thickness decreases. These changes result in an increase in the *C_dl_*. Similarly, an increase of 17.8% in *R_s_* was observed when *S. Typhimurium* cells were binding. The binding of the *S*. *Typhimurium* cells to the electrode surface combined with the insulating properties of the cells’ membrane led to an increase in the resistance at the electrode surface. These attached bacterial cells acted as resistors connected in series with the electrolyte solution resistance in the equivalent circuit. Hence, the increase in the *R_s_* was due to the bound cells’ membrane resistance.

### 3.3. Performance of the Portable Impedance Immunosensing System

**Figure 6:**
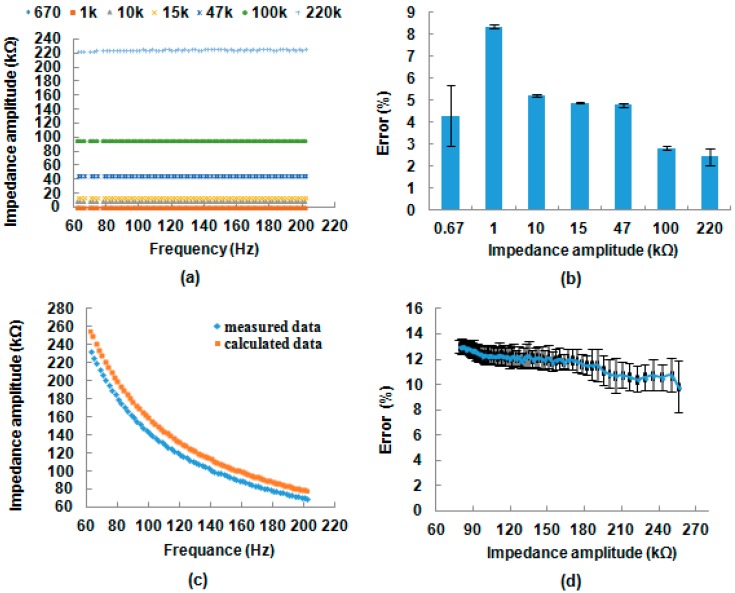
Evaluation of standard impedance elements in the portable impedance immunosensing system, (**a**) mean values; (**b**) error distribution of standard resistors; (**c**) mean values, and (**d**) error distribution of standard capacitors. The means and standard error bars were determined using three replicates.

According to impedance analysis, the best indicator range for impedance spectroscopy between the bacterial and control samples is below 1 kHz. Thus, the impedance spectroscopy in the portable impedance immunosensing system was configured in the frequency range from 60 to 200 Hz. To qualify the performance of detection in the developed system, the feasibility and accuracy of detection for standard resistors and a standard capacitor is shown in Figure 6. Measured resistance values, as shown in Figure 6a, were constant even as the frequency increased. The values were also consistent with the circuit characteristic of pure resistances (Figure 2). As shown in Figure 6b, mean error distributions for the measured standard resistances were not significant, with an error range of 1.4–8.4% and a standard deviation error range of 0.1–1.4%. The measured capacitor impedance distribution (Figure 6c) had a nonlinear relationship and an inverting ratio trend when the frequency increases. It also matched the circuit characteristics of pure capacitors (Figure 2). It demonstrated that the measured capacitance under different frequencies was close to the calculated capacitance. The measured capacitance was compared to the calculated capacitance with an error range of 9.9–13.1% and a standard deviation error range of 0.3–2.1% shown in Figure 6d. These results indicated that the developed system could meet the requirement of impedance detection from an electrical circuit angle.

**Figure 7:**
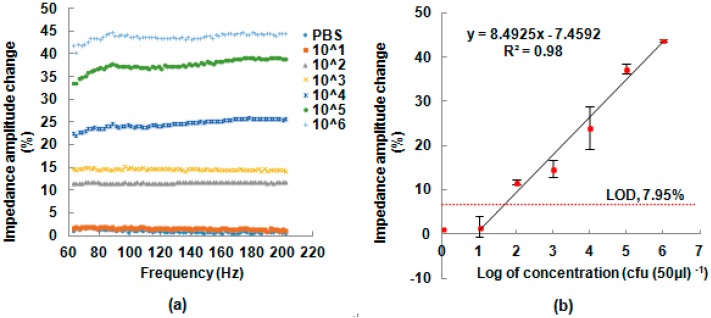
(**a**) impedance change of the negative control sample and concentrations of *S*. *Typhimurium* ranging from 10^1^ to 10^6^ CFU (50 μL)^−1^ in pure culture samples and (**b**) the relationship between the logarithmic value of the concentration of *S*. *Typhimurium* and impedance change at a frequency of 101 Hz. The means and standard error bars were determined using three replications.

To further investigate the ability of the developed system for application in immunosensing detection, the standard resistor and capacitor were replaced by the IDAM based sensor. Figure S1 (provided in Supplemental Materials) shows the impedance spectrum and relevant vibration of the antibody immobilized IDAM sensor after BSA blocking. Detection occurred in the presence of a PBS buffer containing 10 mM [Fe(CN)_6_]^3−/4−^ (1:1). The presence of the redox probe buffer solution resulted in a nonlinear and inverting ratio trend in impedance magnitude compared to the high impedance distribution in the air condition at a frequency range from 60 to 200 Hz. Mean values for detected impedance in the air, as shown in Figure 7a, were distributed from 584.2 to 717.3 kΩ, with a vibration range of 1.0–15.1%. In contrast, the mean values of the redox probe’s detected, as shown in Figure S1b, has a range of 33.9 to 103.6 kΩ, with a vibration range of 6.9–8.8% and the maximum change occurred at 101 Hz. This result demonstrated that the IDAM in the presence of the redox probe buffer solution has the electrical characteristic of a capacitor with respect to being in the air at low frequencies (Figure 4b) and was consistent with the result obtained from a previous study. In addition, the relevant vibration result showed that the impedance detection of the IDAM in the presence of the redox probe buffer solution was more stable than in the air condition. This provides a better condition for bacterial detection.

### 3.4. Detection of S. Typhimurium

The developed immunosensing system was applied for detection of *S. Typhimurium*. Six different bacterial concentrations (76 to 7.6 × 10^6^ CFU (50 μL)^−1^, and expressed in CFU mL^−1^, was 1.52 × 10^3^ to 1.52 × 10^9^ CFU mL^−1^) in pure culture were detected. Figure 7a displays the changes in impedance obtained using the antibody immobilized IDAM at different frequencies and with different concentrations. As it can be seen, there is almost no difference in the impedance change between the negative control samples (PBS buffer solutions) and *S. Typhimurium* solutions at 10^1^ CFU (50 μL)^−1^. There is, however, an obvious difference in the impedance change between 10^2^ and 10^6^ CFU (50 μL)^−1^. A paired *t*-testing was carried out to determine if there was a significant difference between the different *S. Typhimurium* concentrations and impedance change (Table 1). As shown in Table 1, *p*-values of the impedance change of the paired samples were below 0.05 except between the negative control sample (NCs) and 10^1^ CFU (50 μL)^−1^. The results indicate that there is a significant difference between the *S. Typhimurium* in concentrations ranging from 76 to 7.6 × 10^6^ CFU (50 μL)^−1^ (expressed in CFU mL^−1^ was 1.52 × 10^3^ to 1.52 × 10^9^ CFU mL^−1^) and impedance change. This could be due to the increasing number of *S. Typhimurium* cells that bind to the immunosensor’s surface and the increased electron-transfer resistance that results from the binding.

The electrochemical impedance change at 101 Hz caused by the immune reaction at different concentrations of *S. Typhimurium* in pure culture is shown in Figure 7b. There is a linear relationship with a correlation coefficient of 0.98 between the impedance change and the logarithmic value of each *S. Typhimurium* concentration. At a concentration of 10^1^ CFU (50 μL)^−1^, the impedance change of the bacterial sample was close to that of the negative control. The LOD for impedance change of the immunosensor was determined to be 7.95%, which was calculated by the mean of NCs and adding its standard deviation multiplied by three [21,22]. Thus, according to calibration curves obtained by analysis as mentioned, the LOD for *S. Typhimurium* in pure culture was 7.6 × 10^2^ CFU (50 μL)^−1^ (expressed in CFU mL^−1^ was 1.52 × 10^4^ CFU mL^−1^) using the developed impedance platform, which is comparable with other label-free immunosensors for detection of pathogenic bacteria using the commercial electrochemical workstation [31,32,33].

### 3.5. Specificity of the Immunosensor for Detection of Chicken Rinse Water

**Figure 8:**
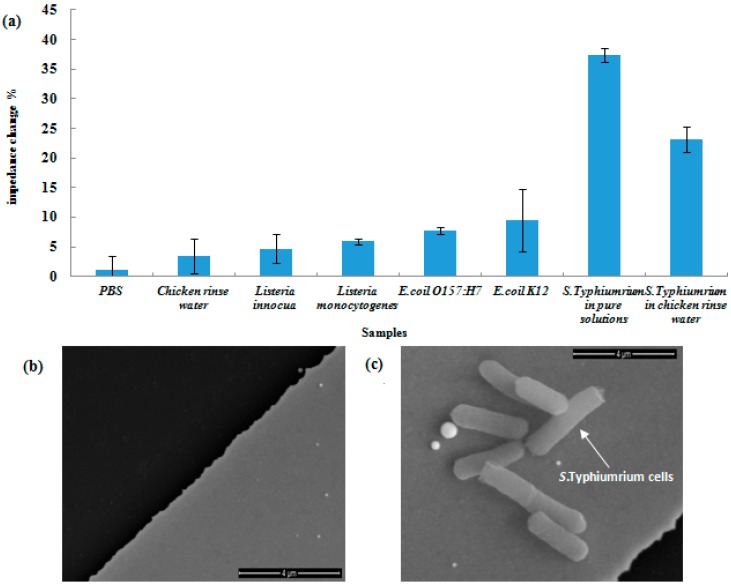
(**a**) Specificity tests of four non-target bacteria and detection of chicken rinse water; (**b**) SEM images of antibodies immobilized IDAM; and (**c**) *S*. *Typhimurium* cells captured by the antibodies which were immobilized on the IDAM surface.

Figure 8a shows the specificity of the immunosensor when different bacteria in pure solutions were detected. (*Listeria innocua*, *Listeria monocytogenes*, *E. coli* O157:H7, *E. coli* K12 and *S. Typhimurium*). There were three samples for each group and the relevant standard deviations are shown as error bars. As shown in Figure 8a, when *S. Typhimurium* and other non-target bacteria were tested using pure cultures at 10^5^ CFU (50 μL)^−1^, only *S. Typhimurium* showed an impedance change of 37.4% and significantly higher than the LOD of 7.9%. Other non-target bacteria yielded negligible impedance changes that were lower or slightly higher than the LOD. In addition, the chicken carcass rinse water experimentally mixed with target *S. Typhimurium* bacteria at 10^5^ CFU (50 μL)^−1^ generated an impedance change of 23.2%. For the chicken carcass rinse water sample, the detected impedance change was lower than the impedance change obtained using the pure culture sample. This was possibly due to the interference of the background of the rinse water. There is an abundant amount of protein, fat and other components presented in chicken carcass rinse water. The combined results indicate that the immunosensor was highly specific for detection of *S. Typhimurium*. Figure 8b is an SEM image that further confirms that *S. Typhimurium* cells were captured on the surface of the antibodies immobilized IDAM. The specificity of the immunosensor was primarily depended on the immobilized anti-*S. Typhimurium* antibodies. The monoclonal anti-*S. Typhimurium* antibodies used in this study could bind to *S. Typhimurium* but not to *L. innocua*, *L. monocytogenes*, *E. coli* O157:H7 and *E. coli* K12.

## 4. Conclusions

In this study, a portable impedance immunosensing system was developed and evaluated for rapid detection of *S. Typhimurium*. The developed portable impedance immunosensing system consisted of a gold interdigitated array microelectrode, a signal acquisitive interface and a laptop computer with LabVIEW software. An equivalent circuit was constructed using the IM-6/Thales 2.49 software to simulate the behavior of the immunosensor. The simulation run using the equivalent circuit showed that the greatest change was found in the electron-transfer resistance and the resistance had an impedance increase of 35.6%. Results from tests using *S. Typhimurium* cells in pure solution showed that there is a linear relationship, with a correlation coefficient of 0.98, between the impedance change and logarithmic value of *S. Typhimurium* cells in concentrations ranging from 7.6 to 7.6 × 10^6^ CFU (50 μL)^−1^. The detection time from the moment a sample was introduced to the display of the results was 1 h and the detection limit was 10^2^ CFU (50 μL)^−1^. The specificity of the immunosensor was validated using four non-target bacteria, and none of them generated detectable signals. In summary, the portable impedance immunosensing system for detection of *S. Typhimurium* is able to achieve an LOD that is comparable with commercial electrochemical impedance instruments. The developed impedance immunosensor has advantages in portability, low cost, rapid detection and label-free features, showing a great potential for in-field detection of foodborne pathogens. Ongoing research focuses on the development of a prototype of the instrument, which consists of a flow cell embedded with an IDME, an automatic sample and reagent delivery unit, a signal conditional interface and a laptop computer with LabVIEW software for rapid detection of *S. Typhimurium* in poultry samples.

## Figures and Tables

**Table 1:** Results of paired samples *t*-tests of the negative control sample and different concentrations of *S*. *Typhimurium* in pure culture samples.

Paired Samples	Mean Difference (%)	Standard Deviations (%)	*p*-Values
NC-10^1^	−0.21	0.41	0.053
10^1^–10^2^	−10.21	0.26	<0.01
10^2^–10^3^	−2.80	0.24	<0.01
10^3^–10^4^	−10.10	1.01	<0.01
10^4^–10^5^	−13.09	0.47	<0.01
10^5^–10^6^	−6.12	0.66	<0.01

NC: negative control samples.

## Supplementary Materials

The following are available online at http://www.mdpi.com/1424-8220/17/9/1973/s1, Table S1: Bacteria culture used in the study; Table S2: Simulated value of *C_dl_*, *R_et_*, and *R_s_* in the equivalent circuit for the negative control and the sample of 2.5 × 10^3^ CFU (50 µL)^−1^ of *S. Typhimurium* and their relative changes percentage with respects to the values of the negative control; Figure S1: A scheme of the portable impedance immunosensing system; Figure S2: Evaluation of the portable impedance immunosensing system using the IDAM sensor under (a) air condition, and (b) PBS buffer solution.
