# Supplementary material for: A Portable Impedance Immunosensing System for Rapid Detection of Salmonella Typhimurium
Source: Sensors (Basel). 2017 Aug 28;17(9):1973. doi: 10.3390/s17091973 (PMC5621372; doi:10.3390/s17091973)
Supplement: Supplementary file 1 [file sensors-17-01973-s001.pdf]

Supplementary Materials:

A Portable Impedance Immunosensing System for Rapid Detection of *Salmonella Typhimurium*

Tao Wen [1,2], Ronghui Wang [2], America Sotero[2] and Yanbin Li [2,*]

[1] School of Mechanical and Electrical Engineering, Central South University of Forestry and Technology, Changsha, Hunan 410004, China; wt207@sina.com

[2] Department of Biological and Agricultural Engineering, University of Arkansas, Fayetteville, AR 72701, USA; rwang@uark.edu (R.W.); asotero@email.uark.edu (A.S.)

* Correspondence: yanbinli@uark.edu; Tel.: +1-479-575-2881

Table S1. Bacteria culture used in the study.

Deposited Name	Designation	Antigenic Properties	ATCC
Salmonella typhimurium (Loeffler) Castellani and Chalmers	CDC 651660	I 4,5,12:i:1,2 (Determined by depositor)	14028
Listeria monocytogenes (Murray et al.) Pirie	1778+ H 2b [KC 1778+ H 2b, KC 1818]	____	43251
Escherichia coli (Migula) Castellani and Chalmers	K12	____	29425
Escherichia coli (Migula) Castellani and Chalmers	CDC B6914MS1	Serotype O157:H7	43888
Listeria innocua Seeliger	SLCC 3379 [58/1971, NCTC 11288]	Serotype 6a	33090

Table S2. Simulated value of C_{dl}, R_{et}, and R_s in the equivalent circuit for the negative control and the sample of 2.5×10^3 CFU (50 μl)$^{-1}$ of *S.* Typhimurium and their relative changes percentage with respects to the values of the negative control.

Samples	C_{dl} (nF)	R_{et} (kΩ)	R_s (Ω)
Negative control	1.87 ± 0.18	15.08 ± 0.23	782 ± 53.03
S. Typhimurium	2.11 ± 0.35	20.45 ± 0.39	921 ± 81.24
Change (%)	12.83	35.61	17.77

C_{dl}: the double layer capacitance at each electrode; R_{et}: the electron-transfer resistance; R_s: the resistance in medium.

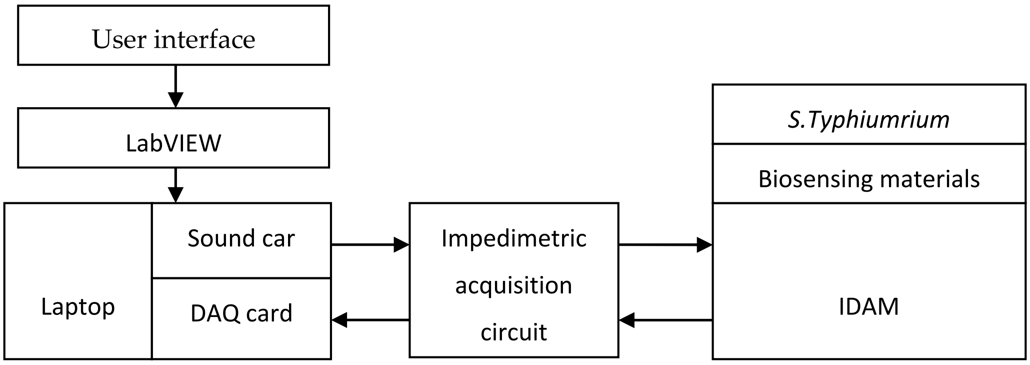

Scheme S1. A scheme of the portable impedance immunosensing system.

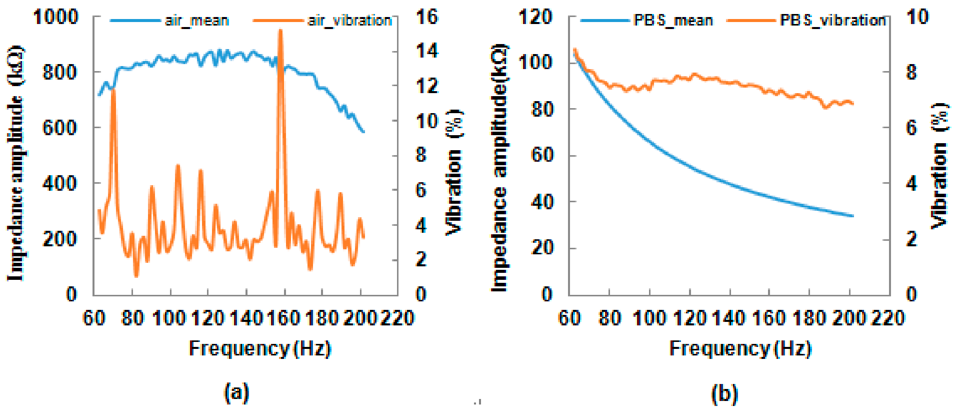

Figure S2. Evaluation of the portable impedance immunosensing system using the IDAM sensor under (**a**) air condition, and (**b**) PBS buffer solution.
